# Biomass Pretreatment and Enzymatic Hydrolysis Dynamics Analysis Based on Particle Size Imaging

**DOI:** 10.1017/S1431927618015143

**Published:** 2018-10

**Authors:** Dimitrios Kapsokalyvas, Arnold Wilbers, Ilco A.L.A. Boogers, Maaike M. Appeldoorn, Mirjam A. Kabel, Joachim Loos, Marc A.M.J. Van Zandvoort

**Affiliations:** 1 Department of Molecular Cell Biology, CARIM, GROW, Maastricht University, Maastricht, Universiteitssingel 50, 6229 ER Maastricht, The Netherlands; 2 Royal DSM N.V., Materials Science Center, Urmonderbaan 22, Geleen 6167 RD, The Netherlands; 3 Royal DSM N.V., Biotechnology Center, Alexander Fleminglaan 1, 2613 AX Delft, The Netherlands; 4 Laboratory of Food Chemistry, Wageningen University, Bornse Weilanden 9, Wageningen 6708 WG, The Netherlands; 5 Department of Molecular Cell Biology, CARIM, GROW, MHeNs, NUTRIM, Maastricht University, Universiteitssingel 50, Maastricht 6229 ER, The Netherlands; 6 Institute for Molecular Cardiovascular Research (IMCAR), RWTH Aachen University, Pauwelstrasse 30, Aachen 52704, Germany

**Keywords:** biomass, enzymatic hydrolysis, large field of view, particle length distribution

## Abstract

Parameters such as pretreatment method, enzyme type and concentration, determine the conversion efficiency of biomass’ cellulose and hemicellulose to glucose and mainly xylose in biomass-based fuel production. Chemical quantification of these processes offers no information on the effect of enzymatic hydrolysis (EH) on particle morphology. We report on the development of a microscopy method for imaging pretreated biomass particles at different EH stages. The method was based on acquiring large field of view images, typically 20×10 mm^2^ containing thousands of particles. Morphology of particles with lengths between 2 *μ*m and 5 mm could be visualized and analyzed. The particle length distribution of corn stover samples, pretreated with increasing amounts of sulfuric acid at different EH stages, was measured. Particle size was shown to be dependent on pretreatment severity and EH time. The methodology developed could offer an alternative method for characterization of EH of biomass for second generation biofuels and visualization of recalcitrant structures.

## Introduction

The production of biofuels from biomass is a field that has developed significantly during the last decade (Ragauskas et al., 2006; Fairley, 2011; Albers et al., 2016). Due to environmental, economic, and social concerns (Kleiner, 2008) regarding the use of edible products for use in the industry or energy sector, the focus has diverted on the use of residue biomass to produce bioethanol and other chemicals (Ragauskas et al., 2006; Fairley, 2011). This second-generation biofuels are commonly produced from corn stover, sugar cane bagasse, or woody biomass from forest or industrial by-products (Ragauskas et al., 2006). The raw material for producing this energy form is considered sustainable, renewable, and it is readily available, without the need of “dirty” extraction methods compared to fossil fuels. However, the extraction and conversion of cellulose and hemicellulose, which are the energy bearing molecules in biofuel production, is a complex process associated with considerable costs.

Cellulose is well-protected in a thick plant cell wall and pretreatment processes are used to break-up the lignin and/or xylan bonds in the wall (Behera et al., 2014). This step of pretreatment is an active field of investigation and several methods have been developed such as hot water, dilute acid pretreatment, and lime (Wyman, 1994; Zhao et al., 2012; Behera et al., 2014). The choice of pretreatment depends on the biomass type, and on the efficiency of the pretreatment to “liberate” cellulose and hemicellulose in a speedy and cost-efficient way, without destroying it. In this respect investigation on the different parameters such as temperature, pressure, or concentration of chemicals is necessary for optimizing the overall process (Zhao et al., 2012). After cellulose and hemicellulose are made accessible in the process of pretreatment, the process of enzymatic hydrolysis (EH) follows. In this step, specialized enzymes convert cellulose to glucose and hemicellulose to mainly xylose (depending on the hemicellulosic structure other sugars are released as well) (Avci et al., 2013). The choice of enzyme mixture, consisting of cellulases and hemicellulases depends on the biomass type and is crucial for the conversion to sugars (Banerjee et al., 2010). Afterwards, a yeast fermentation converts glucose (and depending on the yeast xylose) to ethanol and, finally, distillation is used to purify the final product. For a widespread use of biofuels, their production has to be at least as economic as that of fossil fuels, therefore the whole downstream process has to be optimized (Viikari et al., 2012; Albers et al., 2016).

Optimization of these processes includes cost efficiently maximizing the enzymatic conversion yields of cellulose and hemicellulose to glucose and xylose. Conversion efficiency measurements are performed with techniques such as high performance liquid chromatography (HPLC) (Cheng et al., 2010; Foston & Ragauskas, 2012; Liu et al., 2012), nuclear magnetic resonance (Hu et al., 2011; Foston et al., 2012), or near infrared spectroscopy (Hames et al., 2003; Templeton et al., 2009). The principle behind such applications is that, if the conversion efficiency is increasing, then the optimization steps followed in the pretreatment or EH process were successful. Such an approach, focuses exclusively on the chemical composition of the sample, ignoring the effect of morphological changes. Pretreatment modifies significantly the plant cell wall morphology, which will largely determine the accessibility of enzymes to cellulose and hemicellulose during EH. This will ultimately define the performance and yield of the overall process. Furthermore, EH itself also changes the morphology of biomass particles. Therefore, understanding the dynamics and influence of these processes on biomass particle morphology could offer valuable insight on their mechanism of action and their capabilities regarding efficiency.

In this study, the focus was on imaging, image processing, and statistical analysis, of biomass samples at different pretreatments and EH stages. Special consideration was given to imaging large amounts of individual particles to reveal particle length distributions (PLD). Biomass samples are characterized by particles with high dynamic range in size. Most of the particle size characterization techniques rely on light scattering methods (Jager et al., 2011) which are high-throughput and efficient. However, visual information on individual particles can only be achieved with imaging techniques. Optical (brightfield) microscopy, can offer visual evidence of individual particle morphology regarding shape, as well as plant cell wall morphology. Moreover, it is not limited in dynamic range as any particle size can be visualized and measured. Therefore, it is most accurate in this respect. In conventional microscopy, the number of particles that can be imaged in one image is limited by the field of view of each image, however it can be further extended with stitching techniques. This can be accomplished by acquiring numerous adjacent images of the sample and stitching them together to create one large field of view (LFOV) image in the order of hundreds of square millimeters. The stitching method has already several applications (Madabhushi et al., 2014; Penzias et al., 2016). On such LFOV images, small (in the order of *μ*m) and large particles (in the order of mm) can be well visualized, without loss in resolution, and quantified. In this way, millions of particles can be visualized and characterized, making the whole sample characterization possible in a time-efficient manner.

The goal of this study was to investigate the capabilities of the LFOV imaging methodology in the field of biomass bioconversion and to develop methodologies for analyzing such images. Corn stover samples that received different dilute acid pretreatments and at different stages of EH were imaged and analyzed with focus on the microscopic morphological changes, and on particle size distribution dynamics due to pretreatment and during EH.

## Materials and Methods

### Biomass Samples

Corn stover samples (Table 1)

**Table 1:** Sample Information Regarding Pretreatment and Enzymatic Hydrolysis Parameters.

Sample	Pretreatment conditions	Hydrolysis time (h)
S1-0		0
S1-16	0.36 w/w% H_2_SO_4_, pH 2.3, 1 min	16
S1-120		120
S2-0		0
S2-16	0.60 w/w% H_2_SO_4_, pH 1.9, 1 min	16
S2-120		120
S3-0		0
S3-16	1.44 w/w% H_2_SO_4_, pH 1.3, 1 min	16
S3-120		120

were collected and pretreated based on National Renewable Energy Laboratory guidelines, with varying concentration of H_2_SO_4_, namely 0.36, 0.60 and 1.44 w/w%, at 190°C for 1 min holding time. Pretreated samples were diluted to a dry matter concentration of 15 w/w% in a 1 L reactor and incubated at 62°C and pH 4.5 under continuous stirring with DSM enzymes containing cellulases and hemicellulases (DSM, Delft, The Netherlands). Enzymes were dosed in such a way that considerable glucan conversion was reached. After 0, 16, and 120 h samples were taken, heated for 30 min at 95°C to inactivate the enzymes. Samples were stored at 4°C until further analysis. For imaging, samples were diluted with distilled water at 10 *μ*g dry mass/mL. At this concentration particle density was low enough for visualizing each particle. Samples were mounted on a microscope slide and covered with a coverslip. The sugar composition in the pretreated corn stover and enzyme hydrolysates was measured according to a method described elsewhere (De Souza et al., 2013).

### Imaging–Large Field of View

LFOV images were acquired with a Keyence VHX 5000 (Keyence Corporation, Itasca, IL, USA) operated in stitching mode. Images were acquired with 200× magnification (lens model VH-Z20R; Keyence Corporation). The microscope’s resolution performance is detector limited, which means that the resolution is limited by the detector’s pixel size and not by the optics. Therefore, the resolution of the system in the 200× magnification setting is defined by the field of view of each pixel of the complementary metal-oxide-semiconductor sensor, which is 1.1 *μ*m. Each image tile acquired had 1,600×1,200 pixel resolution which corresponded to a 1.7×1.3 mm^2^ FOV. A series of 17×11 image tiles were acquired and subsequently stitched together to produce the LFOV image. Images had 30% overlap to allow for good stitching. The resulting LFOV image had a field of view of 20×10 mm^2^. Acquisition of image tiles and subsequent stitching was performed automatically by the microscope software. Since this is a widefield approach, acquisition of each image tile depends on the camera exposure, which is in the ms range. Acquisition of all the images, including stage translation, requires 3 min approximately, and stitching and saving the LFOV image 3 min approximately. In all, three LFOV images for each sample condition were acquired and analyzed. The resolving power of the microscope, therefore also the minimum particle length that could be measured, was 1.1 *μ*m (determined by the pixel size) and the maximum measurable particle length was 22.3 mm (defined by the diagonal of the LFOV image). However, since a four-pixel cut-off (any object with pixel size below the cut-off value is ignored) is applied during image analysis (see section below) the minimum measured particle length was 2.2 *μ*m (2×2 pixel configuration).

### Particle Size Analysis

LFOV images were processed with a median filter to remove noise and segmented based on intensity thresholding. Afterwards each particle’s cross sectional surface was measured with the analyze particles plugin of ImageJ (Schneider et al., 2012). A four-pixel cut-off was used to eliminate any noise artifacts. Most particles appeared with an elongated cylinder like morphology. An ellipsoid was fitted to each particle and its major and a minor axis were calculated. The major axis of the ellipsoid was considered the effective particle length, which from now on will be simply called particle length. PLD histograms were reconstructed based on particle surface weighting. Particle surface, instead of particle count, was used as weighting. This method was chosen, since there are many small particles compared to the number of large particles. However, these small particles contribute less to the overall mass of the sample. Therefore, particle count weighting would result in an underestimation of average particle size. PLD were calculated with a custom routine in MATLAB 7.1 (Mathworks, Natick, MA, USA). PLD were fitted with a bi-lognormal curve with the curve fitting tool of MATLAB. The fitting parameters of the bi-lognormal distribution were *ℓ*_1,_ and *ℓ*_2_, *a*_1_ and *a*_2_, *w*_1_ and *w*_2_ which correspond to the center, the area, and the log standard deviation of each lognormal fitted curve. A nonlinear least square method was used with the Trust-Region algorithm and the bisquare robust regression method. The probability of each length bin was used as fitting weight. Average particle length (<*ℓ*>) was calculated from the curve fitting results as1
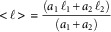
where the denominator serves as normalization. The contribution of small particles population to the total sample can be extracted by the ratio of *a*_1_ over the sum of *a*_1_ and *a*_2_. For each sample, hundreds of thousands of particles were imaged and analyzed (Table 2) to provide statistically-significant results.

**Table 2:** Particle Length Distribution Parameter Fitting Results.

Sample	*a*_1_ (minor peak parameter)	*ℓ*_1_ (*μ*m) minor peak	*a*_2_ (minor peak parameter)	*ℓ*_2_ (*μ*m) major peak	<*ℓ*> (*μ*m) average length	Number of particles (1,000)	Goodness of fit (*R* ^2^)
S1-0	0.43	18	126	2,120	2,113	400	0.95
S1-16	0.68	26	17	311	300	370	0.94
S1-120	0.32	14	6	209	204	430	0.89
S2-0	0.37	20	88	1,375	1,370	425	0.97
S2-16	1.20	35	10	251	229	475	0.97
S2-120	0.76	25	3	140	119	480	0.97
S3-0	0.56	21	43	639	632	470	0.96
S3-16	0.49	21	6	179	167	365	0.91
S3-120	0.92	25	4	135	117	580	0.98

## Results

### Particle Length Analysis

The LFOV methodology was used to visualize the morphology of pretreated and hydrolyzed corn stover samples. It is based on brightfield microscopy, therefore contrast comes from absorption of light from biomass particles. Particles appear with colors ranging from light to dark brown, indicating that particles absorb in the full visible spectrum. Their brightness is inversely proportional to the thickness of the sample. Moreover, samples are diluted enough so each individual particle can be clearly visualized. The LFOV technique offers the possibility of imaging small particles (~*μ*m) and many large particles (~mm) in the same image with good resolution. Thus, a broad dynamic range of particle sizes can be visualized in one image. Since such images are representative of the sample population, they can be used to quantify PLD of each sample. Particle length quantification can be performed by image segmentation and subsequent particle size analysis.

**Figure 1:**
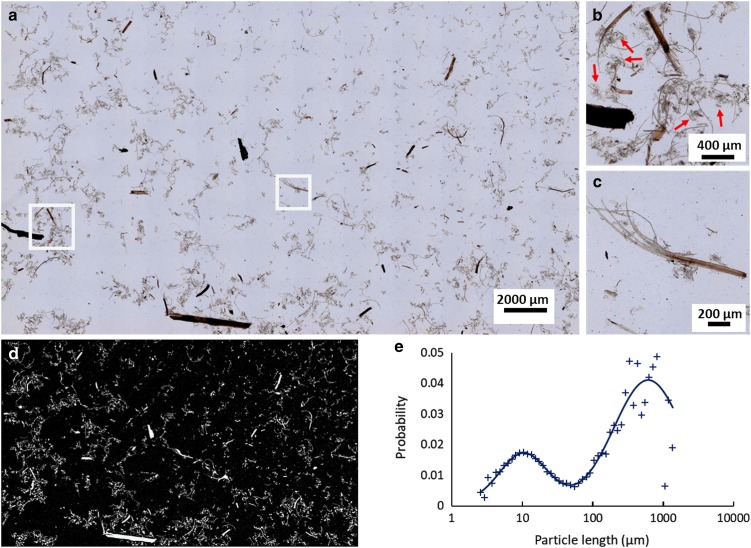
Particle size analysis based on the large field of view (LFOV) methodology. **a**: Characteristic LFOV image of sample S1-0 with a field of view 27×16 mm. **b**: Magnified region of Figure 1a. Particles with long strings and particle complexes (red arrows) attached to them, are visible. **c**: Magnified inset of (**a**). A particle which appears intact on the right side, and on the other end is degrading to components. **d**: Segmented image of Figure 1a. Based on this image particle size is calculated. **e**: Particle size distribution of Figure 1b, data points (cross) and the resulting bi-lognormal fitting (line). Two peaks are visible, a major peak at 3,476 *μ*m particle length and a minor at 32 *μ*m.

A representative case is illustrated in Figure 1. The LFOV image of sample S1-0 is presented in Figure 1a, where large and small particles can be discerned. A 3-mm long particle can be clearly seen at the bottom of the image. Smaller particles are better visible in the magnified images of the insets in Figure 1a and are presented in Figures 1b and 1c. In Figure 1b, both large and small particles are visible. Particles appear to have elongated morphology with varying lengths. Also, smaller particle complexes (red arrow, Fig. 1b) appear to be attaching to long particle strings. In Figure 1c, the degrading effect of pretreatment is clearly visible. One end of the particle is thick and uncompromised, while the other end shows detachment of individual tissue components. There, the particle seems to be decomposing into individual, smaller particles. Details of particle morphology are better visible in such images and resolution is adequate to perform accurate particle length quantification. Good image resolution is required for visualizing the products of pretreatment and hydrolysis, since both processes result in smaller particles.

To calculate the average particle size of each sample, the dimensions of each particle have to be measured. An example of this procedure is presented in Figure 1d. A simple intensity thresholding is applied in the image of Figure 1a and it is converted to the binary image of Figure 1d. In this segmented image, the size of each particle can be quantified using the particle analysis routine. The corresponding surface-weighted histogram of particle lengths of Figure 1a is presented in the graph of Figure 1e (cross). The histogram is fitted with a bi-lognormal curve to produce the PLD and is presented with a continuous line in the graph of Figure 1e. In this graph, two peaks can be distinguished, both representing the average length of the distribution of a subset of particle populations, that is, large and small particles. The area parameters *a*_1_ and *a*_2_ correspond to the respective population weight contribution to the overall curve. Analysis of these parameters and position of the PLD peaks can provide statistically relevant information on the dynamics of particle size evolution dependent on sample treatment. In the case of S1-0, two main peaks are identified, namely a major peak at 2,120 *μ*m and a minor at 19 *μ*m. Based on the contribution of each lognormal distribution (parameters *a*_1_ and *a*_2_) the average particle length is calculated, according to equation (1), to be about 2,113 *μ*m. The population of large particles contributes more than 99% to the total PLD curve, also reflected by the average length being so close to the major peak value.

With this methodology, particle length statistics for each sample can be quantified, by analyzing many images from each condition, and the profile of the distributions for the whole sample can be reconstructed and further analyzed. The position and the height of the peaks of these graphs also provide information on the dynamics of particle size. The major peak defines which population of particles is dominant and the distance of the peaks gives evidence on how different are the populations of large and small particles.

### Particle Length Quantification after Pretreatment

**Figure 2:**
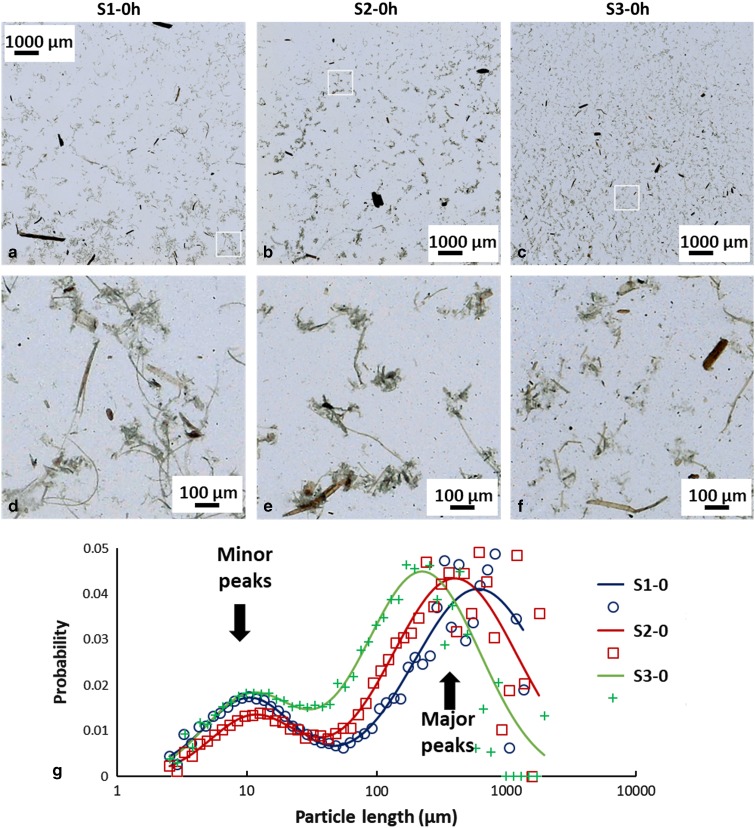
Large field of view (FOV) images of differently pretreated biomass samples. **a**: Low acidic pretreatment, S1-0, (**b**) medium acidic pretreatment, S2-0, (**c**) high acidic pretreatment (S3-0). **a**–**c**: 10×10 mm FOV, cropped images are used to present with higher detail the overall appearance of the samples, (**d**–**f**) magnified regions of above images (**a**–**c**) with 1×1 mm FOV. **g**: Normalized particle length distributions (PLD) of different pretreatment samples and corresponding fitted curves. Small particle population (minor peak) is almost independent of pretreatment, while large particle population (major peak) shifts to lower values with increasing pretreatment acidity. PLD data points are indicted by crosses (S1-0), squares (S2-0) and circles (S3-0), solid lines indicate PLD fitted curves.

Representative LFOV images from pretreated samples with 0.3% (S1-0), 0.6 % (S2-0), and 1.4% (S3-0) sulfuric acid are seen in Figure 2. Corresponding magnified insets from each case are presented in Figures 2d–2f. In such images, the characteristic appearance of each sample becomes better visible. In S1-0, it is common to find particles that are some mm long, however in higher acidic pretreatments (S2-0, S3-0) the maximum particle size observed decreases (to the range of 1 mm or less). Apart from maximum particle length, also morphological changes are seen in smaller particles. In S3-0, many small particles in the range of a few *μ*m are visible, whereas in S1-0 such particles are not as common. Moreover, particle complexes, as indicated in the magnified insets of Figure 1b, appear to change with pretreatment. In S1-0, larger particle complexes with long strings of tissue are commonly visible, whereas these particle complexes appear to reduce in size and frequency with increasing acidity. In S3-0, these complexes are considerably smaller and fewer, while the amount of small (in the range of a few *μ*m) particles is greatly increased.

Quantification of particle length changes is possible with the PLD analysis methodology presented in the previous section. The results are presented in graph Figure 2g and the results of the fitting in Table 2. In the (PLD) curve (Fig. 2g) there is a trend to smaller lengths with increasing acidity, as also observed in the images. In all samples (S1, S2, and S3), two main peaks can be seen. The minor peak represents small particles in the 20 *μ*m range while the major peak represents larger particles in the mm range. In the minor peak, we expect particles that have been chipped off from larger particles during pretreatment. In all cases, this population had a length range between 18 and 21 *μ*m and contributed only weakly to the PLD based on the area parameters *a*_1_ and *a*_2_ (S1-0: 0.3%, S2-0: 0.4%, and S3-0: 1.2%). We conclude that this population is not significantly dependent on pretreatment acidity. On the other hand, the major peak shifts significantly with pretreatment to smaller lengths (S1-0: 2,120 *μ*m, S2-0: 1,375 *μ*m, and S3-0: 639 *μ*m). This large-sized particle population was therefore most affected by the pretreatment procedure. Average particle length in S3-0 (632 *μ*m) was 70% smaller and in S2-0 (1,370 *μ*m) 35% smaller, compared to S1-0 (2,113 *μ*m). The average particle size <*ℓ*> is plotted against increasing acidity in pretreatment in the graph of Figure 4a. This suggests that increasing acidity in the pretreatment will reduce the average particle size of the sample.

Chemical composition analysis (Supplementary Table 1) revealed that insoluble glucan and xylan decreased with increasing sulfuric acid concentration. More specifically, in S2, insoluble glucan was 1.0% less compared to S1 and insoluble xylan 46.8% less. Corresponding values in S3 were 14.0 and 71.8% less compared to S1.

### Particle Length Quantification During Enzymatic Hydrolysis

In the previous section, it was shown that particle size depends on acidic pretreatment. Pretreatment sets a starting point for the following step of EH for each sample. In this section, the effect of EH on these samples is examined. During EH, the molecules of cellulose and hemicellulose are converted to monosugars. This becomes possible after the pretreatment step has weakened the cell wall and created access points for the enzymes to hydrolyze these molecules. Since the building blocks of biomass particles are extracted during EH, particles collapse and break up. This breaking-up is further facilitated by the weakening of lignin bonds during pretreatment and by the stirring inside the EH reactor. Therefore, the dynamics of the EH process are strongly reflected on the particle size of the samples. These dynamics were followed by analyzing PLD.

**Figure 3:**
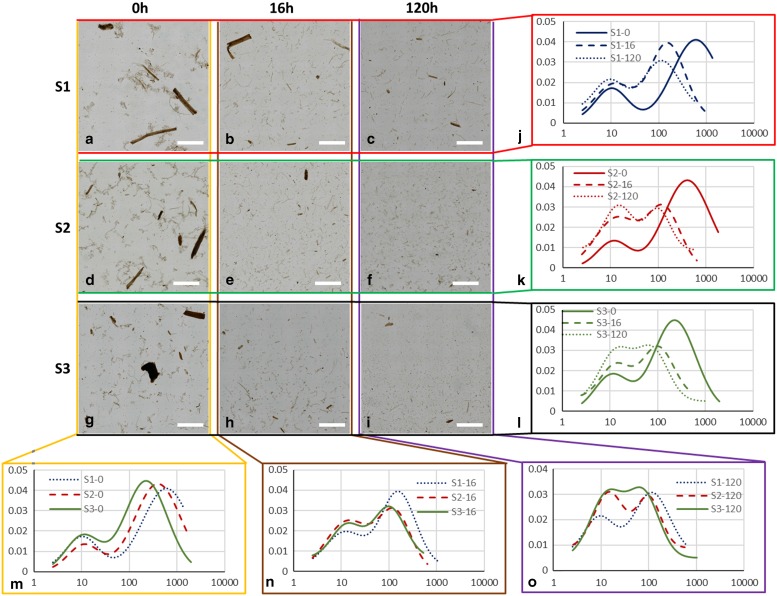
Large field of view (LFOV) images of samples. **a**–**c**: S1, (**d**–**f**) S2, and (**g**–**i**) S3, at 0, 16, and 120 h of enzymatic hydrolysis (EH). Particle length distributions fits for different samples (**j**) S1, (**k**) S2, (**l**) S3, at different EH times (**m**) 0 h, (**n**) 16 h, (**o**) 120 h. Scale bar is 5 mm.

Representative LFOV images of samples S1, S2, and S3 at 0, 16, and 120 h of EH are presented (Fig. 3). Visual observation of these images offers already significant evidence on the degrading effect of EH on particles. In all cases (S1, S2, and S3), particle size is larger in the beginning of EH, while at increasing EH time, particles become smaller. Cross comparison between samples though is very difficult with only visual observations. Therefore, the PLD quantification methodology was employed for following the dynamics of the average particle length in each case.

**Figure 4:**
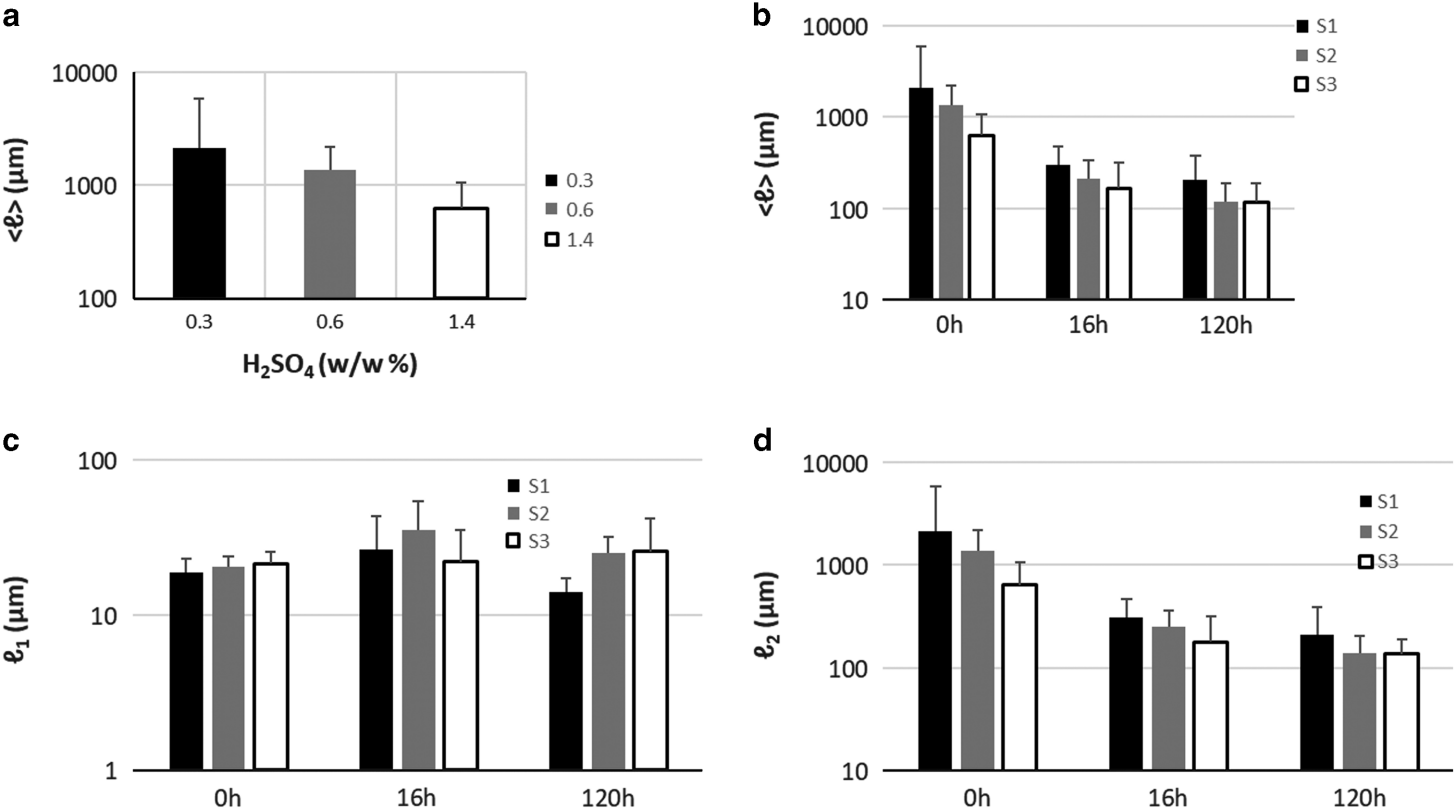
**a**: Particle size versus pretreatment acidity. **b**: Particle size with enzymatic hydrolysis (EH) time, (**c**) *ℓ*_1_ at increasing EH time, (**d**) *ℓ*_2_ at increasing EH time. Error bars represent the confidence bounds.

The normalized PLD are presented in Figure 3. In Table 2 the results of PLD fits are presented. In Figure 2, particle length could be characterized by two populations, large particles (*ℓ*_2_−major peak) and small particles (*ℓ*_1_−minor peak). In Figure 3, the evolution of these populations with EH time is presented. The evolution of particle length based on EH time for each pretreatment is presented in Figures 3j–3l and cross comparison between pretreatments is presented in Figures 3m–3o. In sample S1 (Fig. 3j), the major peak *ℓ*_2_ (2,120 *μ*m at 0 h) decreased 85% at 16 h (311 *μ*m) and further 33% at 120 h (209 *μ*m) leading to a total decrease of 90%. In S2 (Fig. 3k), *ℓ*_2_ (1,375 *μ*m at 0 h) decreased 81% at 16 h (251 *μ*m) and further 44% at 120 h (140 *μ*m), with a total decrease of 89%. In S3, *ℓ*_2_ (639 *μ*m at 0 h) decreased 72% at 16 h (179 *μ*m) and further 24% at 120 h (135 *μ*m), with a total decrease of 78%. The *ℓ*_1_ values changed also with EH, but not monotonically as *ℓ*_2_. In S1 and S2, *ℓ*_1_ initially increased 44% (26 *μ*m) and 75% (35 *μ*m) at 16 h and decreased 46% (14 *μ*m) and 29% (25 *μ*m) at 120 h correspondingly. In S3, there was no change at 16 h, and a 19% increase was observed at 120 h. *ℓ*_1_ and *ℓ*_2_ are presented in the graphs of Figures 4c and 4d.

Apart from particle length, also population size can be investigated by examining the area parameters *a*_1_ and *a*_2_. The contribution of small particles population to the total sample can be extracted by the ratio of *a*_1_ over the sum of *a*_1_ and *a*_2_. In S1, small particles contribution increased from 0.3% at 0 h to 3.8% at 16 h and 5.0% at 120 h. For S2, corresponding contribution was 0.4, 10.7, and 20.2%, and for S3, 1.2, 7.7, and 18.6%. This demonstrates that at the end of EH the small particles population in S1 did not increase to the same degree as in S2 and S3 and this can be also visualized in graphs Figure 3o, where the curves of small and big particles of S2 and S3 tend to fuse at 120 h whereas in S1 large and small particles curves remained well separated and distinct.

All these observations indicate that EH and consequently pretreatment were not as efficient for S1 as it was for S2 and S3, even if the same enzyme cocktail was used. Interestingly, large particles with lengths in the order of mm, were still present at the end of EH (Fig. 3g), indicating that some particles are more recalcitrant to EH. Therefore, these particles do not contribute to the cellulose conversion, limiting the efficiency of the overall process of ethanol production. Future detailed studies on such particles could provide information on the limitation of pretreatment and EH.

Chemical composition analysis on single samples showed that insoluble glucan and xylan declined while glucose and xylose increased during EH (Supplementary Table 2). In S1-120 compared to S1-0 insoluble glucan and xylan decreased 50 and 50%, respectively, while glucose and xylose increased 350 and 80%, respectively. The corresponding values in S2-120 were 60, 60, 390, and 40%. The corresponding values in S3-120 were 70, 50, 350, and 10%.

## Discussion

The gold standard method for characterization and chemical quantification of biomass bioconversion to monosugars is HPLC (Sluiter et al., 2012), since it can provide exact chemical composition information of a sample. Although powerful, it does not provide morphological information of the particles of a sample. Morphological information, acquired through imaging, is important for understanding the process of EH and more importantly for studying recalcitrance, since it has emerged as an important hindering factor in bioconversion (Zeng et al., 2012; Bubner et al., 2013; Karimi & Taherzadeh, 2016). In this study, we pursued to demonstrate that morphological information can be used to not only investigate the local morphology of a particle but also to fully characterize a sample. This was accomplished with the LFOV methodology. We showed that a two-population particle length model is adequate to characterize a biomass sample with good precision. Particle length was shown to be dependent on the severity of pretreatment (Fig. 4a). Particle length was also shown to be dependent on EH time. For all three pretreatment conditions (S1, S2, and S3) particle length shifted significantly to smaller values in the first 16 h of EH, while the size shift for the remaining EH period (120 h) was still significant but at a slower rate. Similar observations have been made on the chemical composition of hydrolyzed corn stover (Zeng et al., 2012; Avci et al., 2013), where based on HPLC, it was shown that conversion of cellulose and hemicellulose to monosugras is faster in the beginning of EH and continues with reduced rate at later stages. Since almost 40% of corn stover is cellulose (Chen et al., 2012; Avci et al., 2013), morphology and chemical composition are related during a process (EH) where most of cellulose is extracted from particles. Change in chemical composition is reflected in morphology and this was quantified by measuring particle length.

Several models have been proposed for describing the effect of pretreatment and EH on the plant cell wall (Chapple et al., 2007; Quiroz-Castañeda & Folch-Mallol, 2013), but the effect on particle size has not been characterized and quantified in detail. In this study, we have visualized morphology and quantified key morphological characteristics of pretreated hydrolyzed corn stover biomass. By examining the evolution of this two-population particle size model we were able to monitor the evolution of particle size under different conditions and compare the results. Particle length decreased significantly in the first 16 h of EH (85.3, 81.75, and 71.9% in S1, S2, and S3 correspondingly) and further 32.7, 44.2, and 24.5% between 16 and 120 h. Based on chemical analysis (Supplementary Table 2) glucan decreased 46.0, 54.2, and 56.4% in S1, S2, and S3, respectively, in the first 16 h of EH and further 15.9, 20.0, and 25.4% between 16 and 120 h. Although, as expected, numbers regarding particle length and glucan content do not match exactly, they do indeed show a very similar trend, a big decrease at initial stages of EH and a smaller decrease at later stages, therefore they are related. What is different regarding particle length and glucan content is that glucan content decreased more in S3 during EH, compared to S1, while particle length decreased more in S1 compared to S3. Since bigger particles are contained in S1, the mechanical stress of stirring in the EH reactor could be greater compared to the smaller particles in S3, therefore a fraction of the particle length decrease could be attributed to mechanical stress and not to particle degradation due to EH. However, it is difficult to decouple the two processes, as particles during EH are degraded and therefore prone to mechanical damage. In addition, length change does not account for total volume change. On the other hand, PLD analysis provides information that is not attainable with chemical composition analysis. Particle length at the end of EH is defined by the severity of pretreatment (Table 2). Particle length provides information on the size of recalcitrant particles. The size of big population particles offers information on how many big particles are still left in the sample with the possibility of being hydrolyzed even further. Optimal EH would be achieved when the two populations large and small particles fuse into a single population, this would indicate that particles cannot be further degraded. In this study, it was shown that in S1-120 the two populations were clearly distinct, therefore leading to increased recalcitrant mass, while in S3-120 the two populations almost fused which is an indication that EH is close to its maximum capacity. Determination of the optimal condition for maximizing the glucose yield while minimizing the severity of pretreatment would require further investigation.

Apart from particle length quantification we were also able to extract qualitative information on particle characteristic morphologies. Most particles appeared with an elongated, almost cylindrical, morphology. After pretreatment, there were several particles showing signs of degradation as one or both ends of the particle were dissociating for the main body. It was also observed that several structures, which we characterized as particle complexes, were present is all sample in the beginning of EH (Figs. 1, 2). The frequency of these particles appeared to be higher in the less severe pretreatment cases. However, a common observation was that these structures were completely absent after 16 h of EH, which is an indication that were easily and fast hydrolyzed. These structures could belong to parts of the plant that have been shown to hydrolyze faster (Zeng et al., 2012), such as the leaves and the pith. At the end of EH small particles were observed in all cases, although in more severe pretreated samples (S3) particle size was smallest.

In this study, we provided a proof of concept of the imaging capabilities of the LFOV method as a tool for biomass characterization. Particle size investigation has been restricted in the analysis of particle length, a feature that is visible in the images acquired. Particle thickness was not investigated because brightfield microscopy has limited axial resolution, thus thickness cannot be measured. Such measurement would be possible with more advanced optical microscopy techniques that offer sectioning capabilities, such as confocal or two-photon microscopy. Information on the particle volume could be then related to particle mass and this information could be directly related to the chemical composition of the sample. However, such techniques, although powerful, are rather slow, especially when considering that millions of particles need to be analyzed to extract reliable statistics, therefore high throughput investigation would not be practical.

## Conclusions

The developed LFOV methodology was successful in imaging the wide dynamic range of pretreated corn stover particles lengths, and in identifying characteristic structures. Based on the high number of particles analyzed, whole sample characterization was possible with good accuracy. Therefore, this methodology was useful for quantifying the effect of pretreatment on particle size and further able to follow the dynamics of particle size distribution at different stages of EH. The developed methodology could be used to quantify the effect of different pretreatments and EH and use it as a guide for optimization purposes.

## Supplementary material

For supplementary material accompanying this paper visit https://doi.org/10.1017/S1431927618015143. click here to view supplementary material

## References

[ref1] Albers SC, Berklund AM Graff GD (2016) The rise and fall of innovation in biofuels. Nat Biotechnol 34, 814–821. 27504772 10.1038/nbt.3644

[ref2] Avci A, Saha BC, Kennedy GJ Cotta MA (2013) Dilute sulfuric acid pretreatment of corn stover for enzymatic hydrolysis and efficient ethanol production by recombinant *Escherichia coli* FBR5 without detoxification. Bioresour Technol 142, 312–319. 23747442 10.1016/j.biortech.2013.05.002

[ref3] Banerjee G, Scott-Craig JS Walton JD (2010) Improving enzymes for biomass conversion: A basic research perspective. Bioenergy Res 3, 82–92.

[ref4] Behera S, Arora R, Nandhagopal N Kumar S (2014) Importance of chemical pretreatment for bioconversion of lignocellulosic biomass. Renew Sustain Energy Rev 36, 91–106.

[ref5] Bubner P, Plank H Nidetzky B (2013) Visualizing cellulase activity. Biotechnol Bioeng 110, 1529–1549. 23456755 10.1002/bit.24884

[ref6] Chapple C, Ladisch M Meilan R (2007) Loosening lignin’s grip on biofuel production. Nat Biotechnol 25, 746–748. 17621299 10.1038/nbt0707-746

[ref7] Chen Y, Stevens MA, Zhu Y, Holmes J, Moxley G Xu H (2012) Reducing acid in dilute acid pretreatment and the impact on enzymatic saccharification. J Ind Microbiol Biotechnol 39, 691–700. 22167347 10.1007/s10295-011-1068-7

[ref8] Cheng KK, Zhang JA, Chavez E Li JP (2010) Integrated production of xylitol and ethanol using corncob. Appl Microbiol Biotechnol 87, 411–417. 20424835 10.1007/s00253-010-2612-5

[ref9] De Souza AC, Rietkerk T, Selin CGM Lankhorst PP (2013) A robust and universal NMR method for the compositional analysis of polysaccharides. Carbohydr Polym 95, 657–663. 23648027 10.1016/j.carbpol.2013.02.036

[ref10] Fairley P (2011) Next generation biofuels. Nature 474, 9–11. 10.1038/474S02a 21697838

[ref11] Foston M Ragauskas AJ (2012) Biomass characterization: Recent progress in understanding biomass recalcitrance. Ind Biotechnol 8, 191–208.

[ref12] Foston M, Samuel R Ragauskas AJ (2012) ^13^C cell wall enrichment and ionic liquid NMR analysis: Progress towards a high-throughput detailed chemical analysis of the whole plant cell wall. Analyst 137, 3904–3909. 22768393 10.1039/c2an35344j

[ref13] Hames BR, Thomas SR, Sluiter AD, Roth CJ Templeton DW (2003) Rapid biomass analysis: New tools for compositional analysis of corn stover feedstocks and process intermediates from ethanol production. Appl Biochem Biotechnol 105–108, 5–16. 10.1385/abab:105:1-3:5 12721471

[ref14] Hu Z, Foston M Ragauskas AJ (2011) Comparative studies on hydrothermal pretreatment and enzymatic saccharification of leaves and internodes of alamo switchgrass. Bioresour Technol 102, 7224–7228. 21571525 10.1016/j.biortech.2011.04.029

[ref15] Jager G, Wulfhorst H, Zeithammel EU, Elinidou E, Spiess AC Buchs J (2011) Screening of cellulases for biofuel production: Online monitoring of the enzymatic hydrolysis of insoluble cellulose using high-throughput scattered light detection. Biotechnol J 6, 74–85. 21184439 10.1002/biot.201000387

[ref16] Karimi K Taherzadeh MJ (2016) A critical review of analytical methods in pretreatment of lignocelluloses: Composition, imaging, and crystallinity. Bioresour Technol 200, 1008–1018. 26614225 10.1016/j.biortech.2015.11.022

[ref17] Kleiner K (2008) The backlash against biofuels. Nat Rep Clim Change 2, 9–11.

[ref18] Liu X, Ai N, Zhang H, Lu M, Ji D, Yu F Ji J (2012) Quantification of glucose, xylose, arabinose, furfural, and HMF in corncob hydrolysate by HPLC-PDA-ELSD. Carbohydr Res 353, 111–114. 22516168 10.1016/j.carres.2012.03.029

[ref19] Madabhushi A, Toth R, Shih N, Tomaszewski J, Feldman M, Kutter O, Yu D, Paulus J Paladini G (2014) Histostitcher™: An informatics software platform for reconstructing whole-mount prostate histology using the extensible imaging platform framework. J Pathol Inform 5, 8. 24843820 10.4103/2153-3539.129441 PMC4023035

[ref20] Penzias G, Janowczyk A, Singanamalli A, Rusu M, Shih N, Feldman M, Stricker PD, Delprado W, Tiwari S, Böhm M, Haynes A-M, Ponsky L, Viswanath S Madabhushi A (2016) AutoStitcher: An automated program for efficient and robust reconstruction of digitized whole histological sections from tissue fragments. Sci Rep 6, 29906. 27457670 10.1038/srep29906 PMC4960603

[ref21] Quiroz-Castañeda RE Folch-Mallol JL (2013) Hydrolysis of Biomass Mediated by Cellulases for the Production of Sugars In: Sustainable Degradation of Lignocellulosic Biomass—Techniques, Applications and Commercialization, Chandel AK and da Silva SS (Eds.), pp. 119–155. London: InTech.

[ref22] Ragauskas AJ, Williams CK, Davison BH, Britovsek G, Cairney J, Eckert CA, Frederick WJ, Hallett JP, Leak DJ, Liotta CL, Mielenz JR, Murphy R, Templer R Tschaplinski T (2006) The path forward for biofuels and biomaterials. Science 311, 484–489. 16439654 10.1126/science.1114736

[ref23] Schneider CA, Rasband WS Eliceiri KW (2012) NIH image to ImageJ: 25 years of image analysis. Nat Methods 9, 671–675. 22930834 10.1038/nmeth.2089 PMC5554542

[ref24] Sluiter A, Hames B, Ruiz R, Scarlata C, Sluiter J, Templeton D Crocker D (2012) Determination of structural carbohydrates and lignin in Biomass. Technical Report NREL/TO-510-42618.

[ref25] Templeton DW, Sluiter AD, Hayward TK, Hames BR Thomas SR (2009) Assessing corn stover composition and sources of variability via NIRS. Cellulose 16, 621–639.

[ref26] Viikari L, Vehmaanperä J Koivula A (2012) Lignocellulosic ethanol: From science to industry. Biomass Bioenerg 46, 13–24.

[ref27] Wyman CE (1994) Ethanol from lignocellulosic biomass – Technology, economics, and opportunities. Bioresour Technol 50, 3–16.

[ref28] Zeng M, Ximenes E, Ladisch MR, Mosier NS, Vermerris W, Huang CP Sherman DM (2012) Tissue-specific biomass recalcitrance in corn stover pretreated with liquid hot-water: SEM imaging (part 2). Biotechnol Bioeng 109, 398–404. 21928340 10.1002/bit.23335

[ref29] Zhao X, Zhang L Liu D (2012) Biomass recalcitrance. Part II: Fundamentals of different pre-treatments to increase the enzymatic digestibility of lignocellulose. Biofuels Bioprod Biorefin 6, 561–579.

