# Supplementary material for: Biomass Pretreatment and Enzymatic Hydrolysis Dynamics Analysis Based on Particle Size Imaging
Source: Microsc Microanal. 2018 Oct;24(5):517–25. doi: 10.1017/S1431927618015143 (PMC6378656; doi:10.1017/S1431927618015143)
Supplement: Supplementary file 1 [file S1431927618015143sup001.docx]

**Supplementary Material**

**Biomass pre-treatment and enzymatic hydrolysis dynamics analysis based on particle size imaging**

Dimitrios Kapsokalyvas^*^, Arnold Wilbers, Ilco A.L.A. Boogers, Maaike M. Appeldoorn, Mirjam A. Kabel, Joachim Loos, Marc A.M.J. Van Zandvoort

**1 Chemical composition analysis of sulfuric acid treated corn stover.**

The chemical analysis results based on a method described elsewhere (De Souza et al., 2013) of different pre-treatments (S1-0, S2-0, S3-0) are presented in Table S1. The values are expressed as % of dry mass (DM). Data presented are averages ± standard deviations of triplicates. Insoluble glucan and xylan decreased with increasing sulfuric acid concentration. In S2, insoluble glucan was 1.0% less compared to S1 and insoluble xylan 46.8% less. Corresponding values in S3 were 14.0 % and 71.8% less compared to S1. Glucose and xylose increased with increasing pre-treatment severity. Glucose was 40.0% in S2 and 107.1% higher in S3 compared to S1. Xylose was more than 2 times higher in S2 and 4.5 times higher in S3 compared to S1.

**Table S1**: Chemical composition analysis of S1, S2, and S3 pre-treated corn stover samples expressed as % of DM.

| **Sample** | **total glucan** | **insoluble glucan** | **glucose*** | **total xylan** | **insoluble xylan** | **xylose*** |
| --- | --- | --- | --- | --- | --- | --- |
| **S1-0** | 40.1 ± 0.8 | 37.8 ± 0.6 | 1.0 ± 0.1 | 14.0 ± 0.4 | 6.4 ± 0.7 | 2.7 ± 0.1 |
| **S2-0** | 39.3 ± 0.3 | 37.4 ± 0.4 | 1.4 ± 0.0 | 12.9 ± 0.3 | 3.4 ± 0.2 | 6.0 ± 0.2 |
| **S3-0** | 36.5 ± 0.5 | 32.5 ± 0.3 | 2.9 ± 0.2 | 14.4 ± 0.4 | 1.8 ± 0.1 | 12.2 ± 1.0 |

* expressed as polymer (glucose/(180/162), xylose/(150/132), Mw glucose = 180 Da -H2O (when present as oligo/polymer) =162, mW xylose=150Da - H2O(when present as oligo/polymer) =132.

**2 Composition analysis of pre-treated corn stover and enzyme hydrolysates**

Chemical composition analysis based on a method described elsewhere (De Souza et al., 2013) was performed on single samples of differently pre-treated corn stover during enzymatic hydrolysis. Results are presented on Table S1. The amount of insoluble glucan and xylose are pre-treatment dependent. At increasing H_2_SO_4_ concentration both insoluble glucan (S1-0:38.9, S2-0: 38.4, S3-0: 35.2) and xylan (S1-0:4.5, S2-0: 4.1, S3-0: 1.3) decreased. Insoluble glucan and xylan further decreased at increasing enzymatic hydrolysis time, while glucose and xylose increased. In S1-120 compared to S1-0 insoluble glucan and xylan decreased 50% and 50% respectively, while glucose and xylose increased 350% and 80% respectively. The corresponding values in S2-120 were 60%, 60% 390%, and 40%. The corresponding values in S3-120 were 70%, 50%, 350%, and 10%.

Table S2: Chemical composition measured in enzyme hydrolysates expressed as % of DM.

| **Sample** | **total glucan** | **insoluble glucan** | **glucose*** | **total xylan** | **insoluble xylan** | **xylose*** |
| --- | --- | --- | --- | --- | --- | --- |
| **S1-0** | 38.9 | 32.6 | 4.6 | 14.1 | 4.5 | 5.5 |
| **S2-0** | 38.4 | 33.9 | 4.6 | 13.2 | 4.1 | 7.7 |
| **S3-0** | 35.2 | 28.9 | 5.1 | 13.9 | 1.3 | 11.3 |
| **S1-16** | 37.9 | 17.6 | 19.4 | 13.4 | 3.2 | 10.4 |
| **S2-16** | 38.1 | 15.5 | 20.3 | 12.7 | 1.8 | 11.0 |
| **S3-16** | 34.7 | 12.6 | 20.3 | 13.6 | 0.7 | 13.2 |
| **S1-120** | 36.8 | 14.8 | 20.5 | 12.6 | 2.4 | 10.0 |
| **S2-120** | 37.5 | 12.4 | 22.5 | 12.2 | 1.5 | 11.0 |
| **S3-120** | 33.1 | 9.4 | 22.7 | 12.3 | 0.7 | 12.7 |

* expressed as polymer (glucose/(180/162), xylose/(150/132), Mw glucose = 180 Da -H2O (when present as oligo/polymer) =162, mW xylose=150Da - H2O(when present as oligo/polymer) =132.

**References**

De Souza, A.C., Rietkerk, T., Selin, C.G.M., Lankhorst, P.P., 2013. A robust and universal NMR method for the compositional analysis of polysaccharides. Carbohydr. Polym. **95**, 657-663.
